# Optimization of BBR Congestion Control Algorithm Based on Pacing Gain Model

**DOI:** 10.3390/s23094431

**Published:** 2023-04-30

**Authors:** Shuang Yang, Yuquan Tang, Wansu Pan, Huadong Wang, Dandan Rong, Zhirong Zhang

**Affiliations:** 1 Anhui Provincial Key Laboratory of Photonic Devices and Materials, Anhui Institute of Optics and Fine Mechanics, HFIPS, Chinese Academy of Sciences, Hefei 230031, China; shuangy@mail.ustc.edu.cn (S.Y.); laserway@aiofm.ac.cn (Y.T.); wanghd@aiofm.ac.cn (H.W.); rongdan@mail.ustc.edu.cn (D.R.); zhangzr@aiofm.ac.cn (Z.Z.); 2 University of Science and Technology of China, Hefei 230026, China; 3 Guangdong Provincial Key Laboratory of Intelligent Disaster Prevention and Emergency Technologies for Urban Lifeline Engineering, Dongguan 523808, China; 4 Advanced Laser Technology Laboratory of Anhui Province, Hefei 230037, China

**Keywords:** TCP congestion control, BBR, RTT fairness, pacing gain, sending rate

## Abstract

In 2016, Google proposed a congestion control algorithm based on bottleneck bandwidth and round-trip propagation time (BBR). The BBR congestion control algorithm measures the network bottleneck bandwidth and minimum delay in real-time to calculate the bandwidth delay product (BDP) and then adjusts the transmission rate to maximize throughput and minimize latency. However, relevant research reveals that BBR still has issues such as RTT unfairness, high packet loss rate, and deep buffer performance degradation. This article focuses on its most prominent RTT fairness issue as a starting point for optimization research. Using fluid models to describe the data transmission process in BBR congestion control, a fairness optimization strategy based on pacing gain is proposed. Triangular functions, inverse proportional functions, and gamma correction functions are analyzed and selected to construct the pacing gain model, forming three different adjustment functions for adaptive adjustment of the transmission rate. Simulation and real experiments show that the three optimization algorithms significantly improve the fairness and network transmission performance of the original BBR algorithm. In particular, the optimization algorithm that employs the gamma correction function as the gain model exhibits the best stability.

## 1. Introduction

With the continuous expansion of the scale of the Internet, the number of network users and applications is rapidly increasing, and congestion has become an important issue. More congestion control studies have been undertaken to maintain the overall network’s stability [1]. In 2016, Google released a congestion control algorithm (CCA) based on bottleneck bandwidth and round-trip propagation time (BBR) [2]. The BBR algorithm no longer solely uses packet loss or delay as an indicator of network congestion, but instead adjusts the sending behavior of data packets based on measured bottleneck bandwidth (BtlBw) and round-trip propagation time (RTprop). This mechanism does not reduce the congestion window (CWND) caused by a single congestion signal, avoiding a reduction in transmission rate, thereby achieving high throughput while minimizing transmission delay. This is different from traditional congestion control and has ushered in a new era of congestion control.

In various Google implementations, the BBR algorithm has been shown to significantly enhance TCP connection throughput and exhibit advantages over traditional packet loss-based algorithms in this regard [3]. Due to its excellent performance, BBR has received a lot of attention since its release. Mathis and Mahdavi [4] claim that BBR has ushered in a new era of congestion control. However, some issues with the BBR algorithm have been discovered in some BBR evaluation reports [5,6,7,8,9,10]. For example, when BBR shares bottlenecks with Reno/CUBIC, it may lead to unfairness and overly aggressive behavior [5,6,7]; along with high packet loss rates in shallow buffers [8]; and bandwidth injustice across data flows with different round trip time (RTT) [9,10]. To address these issues, numerous academics have proposed optimization schemes [11,12], and Google is actively working to improve these issues. To encourage further optimization, Google has made the BBRv2 alpha version code available [13].

This article aims to provide a comprehensive analysis of the BBR congestion control mechanism, highlighting the algorithm’s performance flaws, with a focus on the most significant RTT fairness issue. Several enhanced models of correlated pacing gain are proposed based on the BBR algorithm’s transmission rate detection mechanism. The performance of the optimized algorithm is tested and verified simultaneously through simulation and experimental research in a natural network environment, helping to advance the BBR algorithm’s development and even the investigation of next-generation CCAs. Additionally, it can guide engineers as they choose appropriate TCP congestion protocols or adjust the parameters in BBR applications.

The rest of this article is arranged as follows. Section 2 outlines the relevant research work on BBR. Section 3 introduces the algorithm of BBR and analyzes RTT fairness issues. Section 4 describes the theoretical derivation of the pacing gain model and the parameter selection process. Section 5 is the real and simulation experimental results and evaluation. Conclusions and discussions are held in Section 6.

## 2. Related Works

Unlike traditional packet loss-based or delay-based CCAs, BBR departs from the conventional approach of utilizing a single congestion signal to reduce transmission rates. Instead, BBR employs two control parameters: pacing rate/sending rate and CWND, enabling dynamic control of transmission rates to achieve high throughput and low latency. The Cardwell team compared the performance of the BBR with the CUBIC algorithm, demonstrating that BBR significantly improves the throughput of TCP connections. Furthermore, Google has integrated BBR into its B4 Wide Area Network (WAN) and YouTube platform, bolstering its practical application. Researchers [14,15,16,17] have conducted optimizations of the BBR algorithm, applying it to various network environments to reduce latency and improve throughput, thus enhancing network performance. As of Linux kernel version 4.19 and later, the congestion control method has been updated from CUBIC to BBR.

With the broad attention devoted to BBR algorithms, numerous studies have evaluated the performance of BBR and investigated its behavior in simulated connections or actual networks [5,18,19,20,21]. Hock et al. [5] assessed the performance of BBR in high-speed bottleneck links for the first time, including RTT fairness, latency, packet loss rate, and fairness coexisting with CUBIC. Ware et al. [18] did a performance analysis of the BBR algorithm and analyzed its advantages. Claypool et al. [19] have conducted numerous experiments on the BBR algorithm and pointed out that excessive BBR evaluation bottleneck bandwidth and RTT lead to protocol unfairness. Casas-Velasco et al. [20] analyze the performance of the CUBIC and BBR protocols in the presence of background traffic. Tao et al. [21] analyzed theoretically and found that the RTT fairness of BBRs is determined by the RTT ratio of the data flow, without involving other network parameters. Scherrer et al. [22] presented a fluid model of BBR, allowing efficient simulation under a wide variety of network settings.

Many scholars have conducted further optimization research to improve its defects. Xie et al. [23] proposed the BBR-Yinker algorithm, which can achieve high throughput and low latency over Wi-Fi and 5G networks by adjusting the pacing_gain of BBR according to the network conditions. Song et al. [24] proposed a BBR congestion window scaling (BBR-CWS) algorithm with packet loss feedback to the size of the CWND based on packet loss feedback, balancing the throughput with other algorithms. Huang et al. [25] put forward an improved method that reduces the RTT and its jitter and improves the convergence speed of the algorithm.

To solve the problem of RTT fairness, Ma et al. [10] proposed a BBQ algorithm. This algorithm avoids delivering too many data packets into the network from long RTT flows by continuously detecting redundant queues. Additionally, it limits the maximum detection time to avoid long RTT flows from preempting short RTT flows in terms of bandwidth. Yang et al. [26] introduced an adaptive BBR algorithm, which improves fairness amongst RTT flows by varying the BBR bandwidth detection period and gain. Njogu et al. [27] proposed an algorithm that adaptively controls the CWND based on buffer queue status computation to alleviate RTT unfairness. Our previous research also proposed an improved adaptive CWND algorithm for RTT fairness in BBR [28].

Research on the BBR algorithm has continued, and Google is also continuously optimizing the BBR algorithm. In IETF-102, a preview version of BBRv2 with open-source code (called BBRv2 alpha) was proposed, encouraging researchers to dig deeper and help evaluate and improve BBRv2. This version improves the fairness of coexistence with loss-based algorithms and reduces the packet loss rate in shallow buffers. In the recent IETF-110 [29], Google proposed BBR. Swift algorithm, which uses delay as a congestion signal to achieve higher fairness and lower retransmission rate. BBRv2 can solve some fairness issues and limitations of the original BBR, but there are still some RTT fairness and intra-protocol fairness issues [22,30,31,32,33]. Kfoury et al. [31] pointed out that BBRv2′s slow ability to identify available bandwidth in network contexts with erratic bandwidth leads to low link utilization. Nandagiri et al. [32] evaluated the differences between the two versions of BBR and found that there are still fairness issues in BBRv2. In addition, BBRv2 deployment in a WAN is a little complicated.

The BBR algorithm still has to be developed, and more work needs to enhance its fairness. When applying the BBR algorithm, exists a potential risk of users exploiting vulnerabilities to engage in bandwidth competition with malicious intent. Although some existing research has improved the fairness of BBR, most of the attention has been paid to inter-protocol fairness. Therefore, new approaches are still required to enhance the RTT fairness of the BBR algorithm and boost the algorithm’s overall functionality.

## 3. BBR Congestion Control Principle

**Figure 1:**
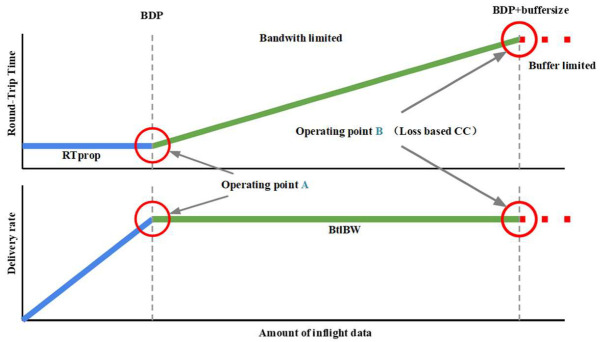
Changes in transfer rate and RTT with inflight: blue lines represent RTprop constraint lines, green lines represent BtlBw constraint lines, and gray dashed lines represent bottleneck buffers.

Traditional loss-based CCA increases the transmission rate by continuously increasing the transmission window (SWND) until packet loss is detected, and then quickly reduces the SWND. The final convergence occurs at the point where packet loss occurs after buffer overflow, as shown in operating point B in Figure 1. However, this approach can only guarantee maximum bandwidth and cannot guarantee delay. It frequently transmits data using the entire link capacity, which can lead to buffer expansion and increased queuing delay, particularly when the buffer is very deep.

In contrast, the BBR algorithm is a novel approach that does not take cache size into account when calculating the bandwidth delay product (BDP) in the BBR model.

At the optimal operation point A in Figure 1, where the amount of data in flight is exactly 1 BDP and the SWND is equal to 1 BDP, the throughput reaches its maximum, and latency is minimized. How to accurately estimate BtlBw and RTprop becomes a key issue. BtlBw and RTprop must be continuously estimated as they can change during the course of a connection. The measurement of RTT in the TCP mechanism is as:

(1) $$RTT_{{}_{t}}=RTprop_{t}+\eta_{t}$$

where *η_t_* represents “noise” introduced by the queue along the path; *RTprop_t_* is the physical attribute of the connection path, which only changes when the path changes. The unbiased estimate of RTprop at time t is:

(2) $$RTprop_{t}=RTprop_{t}+\min(\eta_{t})=\min(RTT_{{}_{t}})\;\forall t\in \left[t-W_{R},\;t\right]$$

where *W_R_* represents the number of minutes (tens of seconds to a few minutes) during which the time window runs.

Unlike RTT, BtlBw is not measured in TCP, but it can be evaluated by measuring the delivery rate. The unbiased estimator of the maximum transfer rate in a window is BtlBw:

(3) $$BtlBw=\;\max\left(deliveryRate_{t}\right)\;\forall t\in \left[t-W_{B};\;t\right]$$

where *deliveryRate_t_* represents the delivery rate, and the *W_B_* is typically 6 to 10 RTTs.

BBR regards the maximum transfer rate in the last 10 RTTs as BtlBw and the minimum delay measured in the last 10 s as RTprop. Based on the measurement model, the BBR controls its transmission behavior through pacing_gain and cwnd_gain, with sending rate and CWND being:

(4) Sending rate=pacing_gain×BtlBw

(5) CWND=cwnd_gain×BtlBw×RTprop

**Figure 2:**
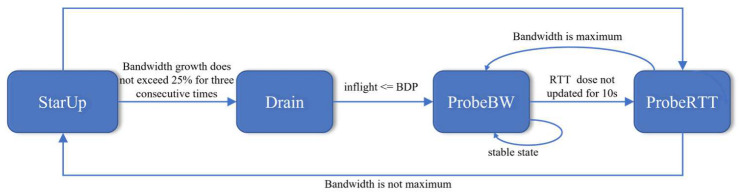
BBR state machine.

The BBR algorithm has four control states: StartUp, Drain, ProbeBW, and ProbeRTT. As shown in Figure 2.

The StartUp state in BBR is very similar to the slow start phase. During this state, both the *pacing_gain* and *cwnd_gain* are set to 2/ln2 (approximately 2.85) to increase their transmission rate and allow the sender to detect the maximum available bandwidth. When the newly estimated BtlBw does not exceed 1.25 times the previous BtlBw estimate for three consecutive times, the algorithm assumes that the link has been filled and the state changes to Drain. In the Drain state, the *pacing_gain* = ln2/2 gradually reduces the transmission rate until the data packet in the link matches 1 BDP, at which point the state changes to ProbeBW.

Most data flows are in the ProbeBW state, with eight pacing gain cycles of different values (*pacing_gain*[] = [1.25; 0:75; 1; 1; 1; 1; 1; 1; 1]), and the duration of each cycle is one RTprop. During the detection rising phase, *pacing_gain* = 1.25 is used to increase the transmission rate and detect more available bandwidth. During the descent phase, *pacing_gain* = 0.75 is used to eliminate excessive queues accumulated. Finally, pacing_gain = 1 is used for six cycles to maintain a stable transmission rate. Meanwhile, the *cwnd_gain* is set to a constant value of 2 to ensure sufficient packets are sent during the probe phase.

If the minimum delay RTprop is not sampled again within 10 s, the BBR mechanism assumes that the link is in a congested state and switches from the ProbeBW state to the next ProbeRTT state. In this state, a data evacuation operation is performed, and the CWND is set to 4MSS. New data packets are injected into the link until the size of the valid data packet is less than 4MSS, and the new minimum delay RTprop value is sampled. After the timeout, the algorithm returns to the StartUp or ProbeBW state based on the network load.

## 4. Optimization Algorithm Based on Pacing Gain Model

### 4.1. Design Motivation

The BBR periodically detects more bandwidth at 1.25 times the BtlBw and queues up excess traffic of 0.25 BDP. Short RTT flows have a smaller estimated BDP than long RTT flows, so their proportion in queue backlog is relatively small, ultimately leading to a decrease in the actual transmission rate. The persistent low transmission rates can cause a decrease in the BtlBw estimate, which further reduces the queue share and initiates a feedback cycle. Long RTT flows have a larger estimated BDP value, so the proportion of persistent queues will increase as the short RTT flows yield, allowing them to transmit data at a higher transmission rate than short RTT flows. Due to the preemption of long RTT flows, it will be difficult for short RTT flows to detect available bandwidth, and their eventual bandwidth tends to be 0.

**Figure 3:**
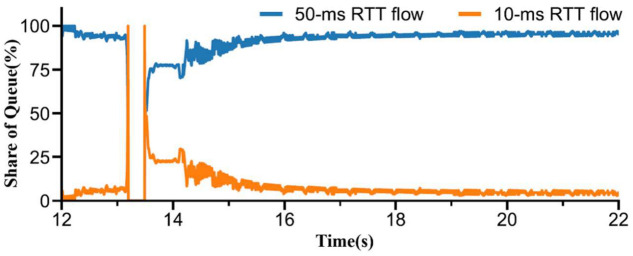
Queue share of two BBR flows from 12 s to 22 s [10].

The throughput of short RTT flows significantly decreases once a queue backlog is formed, while the throughput of long RTT flows slightly decreases. Ma [10] measured the instantaneous queue backlog of 10 ms and 50 ms RTT flows and analyzed its impact on throughput, as depicted in Figure 3. When a competitor (50 ms RTT flow) enters the ProbeRTT state, the 10 ms RTT flow quickly consumes the buffer share, enabling it to detect additional bandwidth. As the queue is emptied by the 10 ms RTT flow and it attempts to identify new bandwidth, the 50 ms RTT flow reverts to the ProbeBW state. The preemption of 50 ms RTT flows poses a challenge to achieving higher transmission rates for short RTT flows. To address the RTT fairness issue in BBR, it is necessary to mitigate the impact of queue backlog on the transmission rate.

In an ideal scenario, the pacing gain coefficient for the ProbeBW state can be calculated based on the absolute value of the transmission rate, replacing the fixed values of 1.25 and 0.75. Different RTT flows can adjust the increase or decrease of the gain coefficient through their respective transmission rates. The above method is not applicable in the actual process since the BBR algorithm measures and calculates the transmission rate on each end-to-end host, making the bandwidth identification between various RTT flows independent.

To ensure fair competition between different RTT flows, the pacing gain of each BBR flow is adaptively adjusted to guide flows to share bandwidth fairly. When the bandwidth ratio of the BBR flow is lower, the upward and downward gains in the ProbeBW state are larger, enabling data flows to rapidly increase throughput during the upward bandwidth detection phase and slowly decrease throughput during the downward emptying phase. Conversely, when the proportion of bandwidth occupied by BBR flows is higher, the upward and downward gains are smaller, ensuring that the flow’s throughput increases slowly during the upward detection phase and decreases rapidly during the downward emptying phase, thereby providing additional bandwidth for other flows.

Based on the above analysis, an improved bandwidth detection strategy is proposed for the ProbeBW state. The original BBR’s fixed pacing gain coefficients of 1.25 and 0.75 are replaced by the pacing gain coefficients *P*_up_ and *P*_down_. If the inflight is less than 1.25 BDP, different RTT flows increase the transmission rate based on the *P*_up_, actively detecting more available bandwidth. As the transmission rate increases, the amount of data in the link gradually exceeds the bottleneck transmission capacity, forming a queue at the bottleneck buffer. If the inflight in the link is greater than 1.25 BDP and packet loss occurs, the transmission rate is reduced based on the *P*_down_, thereby reducing the number of data packets entering the link in the next cycle.

### 4.2. Construction of Pacing Gain Model

#### 4.2.1. Model Derivation

Assume that *n* different flows are passing through a bottleneck link with a bandwidth of C, and *d_i_*(t) denotes the delivered rate of the *flow_i_ I* ∈ [1,*n*] at time t. According to Equation (3), the estimated maximum bandwidth of the data flow at time t can be obtained:

(6) $$BtlBw_{i}\left(t\right)=max\left(d_{i}(t)\right)\left(T\in \left[t-10RTT;\;t\right]\right)$$

In an ideal state, *d*_1_ + *d*_2_ + ⋯ + *d_n_* = C. Let *T_i_*(t) represent the RTT of *flow_i_* at time t, which is determined by:

(7) $$T_{i}\left(t\right)=\frac{q_{i}\left(t\right)}{C}+p_{i}$$

where $q_{i}\left(t\right)/C$ is the queuing delay of *flow_i_* and *p_i_* is the RTprop of *flow_i_*.

Let *I_i_*(t) stand for the inflight, which is the bottleneck link’s upper limit, and be calculated as follows:

(8) $$I_{i}(t)=d_{i}(t)\times T_{i}(t)=d_{i}(t)\times (\frac{q_{i}(t)}{C}+p_{i})$$

During the first upward detection phase, flows can achieve a gain of 1.25 times, so the maximum delivered rate at time *t* is:

(9) $$\max\left(d_{i}(t)\right)=\frac{\max\left(I_{i}(t)\right)}{T_{i}}=\frac{1.25\times p_{i}\times BtlBw_{i}(t-\Delta t)}{T_{i}}$$

The detection period of the BBR is 8RTprop, and the bandwidth estimation of *flow_i_* in the new round is updated to:

(10) $$BtlBw_{i}(t)=\frac{1.25\times p_{i}\times BtlBw_{i}(t-8p_{i})}{T_{i}}$$

The queuing delay is the same for flows that are constrained by the same bottleneck queue. Once a queue is generated, the actual bandwidth cannot be increased by the expected 1.25 times, and the actual gain is $\frac{1.25\times RTprop}{T}$ (less than 1.25). This demonstrates that the effective gain coefficient increases with increasing RTT. The RTT fairness of the BBR algorithm can be improved by adjusting the gain coefficient. So, we attempt to construct a negative feedback model using RTT to constrain the *P*_up_ and *P*_down_. Let *ω* be the percentage of the current RTT and the maximum RTT:

(11) $$\omega_{i}=\frac{T_{i}}{T_{\max}}\;\left(\omega_{i}\in (0,1]\right)$$

where *T*_max_ is the maximum RTT of the flow over this connection, and *ω =* 1 only when the bottleneck link capacity and buffer are fully occupied.

To optimize the BBR algorithm, it is necessary to carefully select the values of Pup and Pdown for the ProbeBW state based on *ω*. For *P*_up_, the correlation function *P*_up_(*ω*) should be a concave curve. When *ω* is large, the *P*_up_(*ω*) should be lowered slowly. When *ω* is small, the *P*_up_(*ω*) should be sensitive to link utilization and rapidly decline. This approach helps to minimize bandwidth usage for high-priority flows while maximizing available bandwidth for lower-priority flows. For *P*_down_, the correlation function *P*_down_(*ω*) should be a convex curve. When ω is large, the *P*_down_(*ω*) should decrease rapidly, and when ω is small, the *P*_down_(*ω*) should decrease slowly. It is important to note that the chosen functions for *P*_up_ and *P*_down_ should be of low complexity, as they will need to be implemented within the BBR algorithm.

#### 4.2.2. Function Selection

To meet the functional requirements outlined in Section 4.2.1, three groups of functions were developed. The first group, which we refer to as BBR-A, uses the inverse proportion function and the cos function to construct the *P*_up_(*ω*) and *P*_down_(*ω*):

(12) $$a=\left\{\begin{array}{l} P_{\mathrm{up}}(\omega_{i})=\frac{2}{\omega_{i}+1}\;\left(P_{\mathrm{up}}(\omega_{i})\in [1,2]\right)\\ P_{\mathrm{down}}(\omega_{i})=\cos(\omega_{i})\;\left(P_{\mathrm{down}}(\omega_{i})\in (0.5,1]\right) \end{array}\right.$$

This approach replaces the fixed pacing gains of 1.25 and 0.75 in the original BBR algorithm with the more flexible *P*_up_ and *P*_down_. BBR-A sets *P*_up_ to between [1,2] to improve the flows detection, while setting *P*_down_ between (0.5,1] to quickly empty the queue.

However, the asymmetric *P*_up_(*ω*) and *P*_down_(*ω*) in the BBR-A algorithm can cause data volume imbalances of 0.5 BDP size on the link, which may lead to queue backlog. To address this issue, a symmetric function *P*_up_(*ω*) and *P*_down_(*ω*) is constructed. Meanwhile, to alleviate the radical detection in BBR-A, the *P*_up_ and *P*_down_ value ranges from [1,1.5] and [0.5,1], respectively. This improved algorithm is abbreviated as BBR-B:

(13) $$b=\left\{\begin{array}{l} P_{\mathrm{up}}(\omega_{i})=\frac{3}{\omega_{i}+2}\;\left(P_{\mathrm{up}}(\omega_{i})\in [1,1.5)\right)\\ P_{\mathrm{down}}(\omega_{i})=\frac{3}{\omega_{i}-3}+2\;\left(P_{\mathrm{down}}(\omega_{i})\in [0.5,1)\right) \end{array}\right.$$

Furthermore, it is discovered that gamma correction is frequently employed in image processing to smooth images when looking for functions. To optimize the pacing gain mode, we combine gamma correction with a pacing gain model, adjusting the gamma correction function parameters to better meet the actual needs of the functions:

(14) $$c=\left\{\begin{array}{l} P_{\mathrm{up}}(\omega )=1.5-0.5\times \omega^{0.25}\;\left(P_{\mathrm{up}}(\omega )\in [1,1.5]\right)\\ P_{\mathrm{down}}(\omega )=1-0.5\times \omega^{4}\;\left(P_{\mathrm{down}}(\omega )\in [0.5,1]\right) \end{array}\right.$$

**Figure 4:**
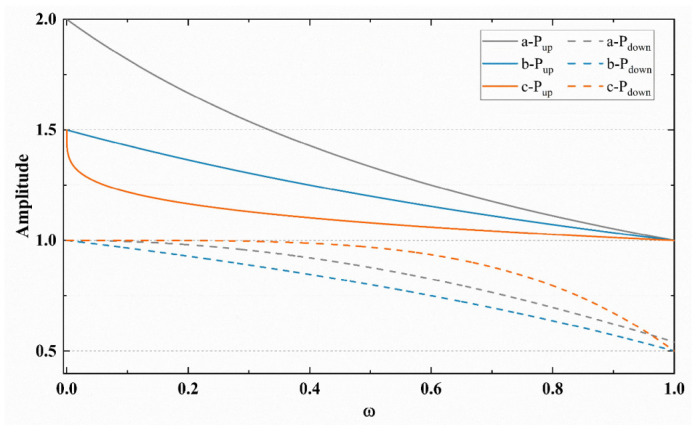
The variation curves of the P_up_ and P_down_ function.

This newly established set of *P*_up_ and *P*_down_ functions forms a new optimization algorithm BBR-C. As shown in Figure 4, the change trends of all three functions meet the set requirements. As *ω* increases, *P*_up_(*ω*) slowly decreases, and the decreasing trend becomes slower. For *P*_down_(*ω*), as ω increases, *P*_down_(*ω*) slowly decreases, but the downward trend accelerates. The *P*_up_ and *P*_down_ built using gamma correction among them more closely adhere to the variation rule of ideal pacing gain.

## 5. Performance Evaluation

**Figure 5:**
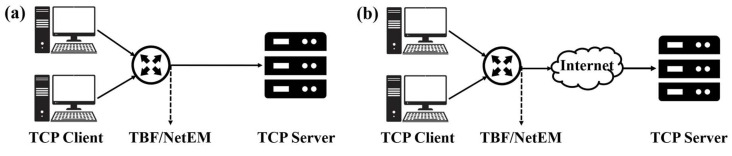
Real network test platform topology: (**a**) LAN testing; (**b**) WAN testing.

To evaluate the performance of the CCAs, we constructed small-scale real and a variety of simulated network scenarios using cloud servers and NS3. We compared the performance of BBR-A, BBR-B, and BBR-C algorithms with the original BBR, BBR variant algorithms BBQ, and BBRv2. In the small-scale real network environment, we deployed four Linux hosts and four cloud servers located in Hefei, Beijing, Hong Kong, and Singapore. We conducted multiple experiments to assess the effectiveness and stability of each algorithm under different scenarios. The entire test environment is divided into LAN and WAN. Using NS3, we also simulated various network scenarios, including different RTTs, buffer sizes, and flows. Through these experiments, we were able to determine the strengths and weaknesses of each algorithm. The topology diagram is given in Figure 5.

### 5.1. Network Performance under Different Buffer Sizes

**Figure 6:**
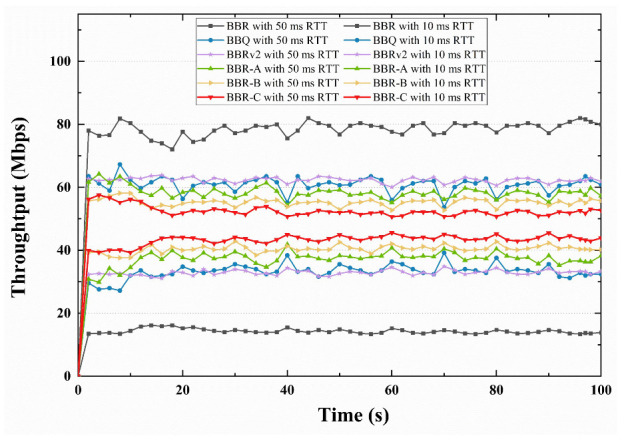
Throughput comparison of different congestion algorithms for 10 ms RTT flows and 50 ms RTT flows in competition.

First, the fairness of various optimization strategies is examined along with the throughput differences of various RTT flows in the same connection using NS3. The experimental setup used a bottleneck bandwidth of 100 Mbps and a buffer size of 5 BDP. Figure 6 displays the throughput statistics for the 10 ms and the 50 ms RTT flow for the six algorithms. The throughput of a 50 ms RTT flow for the BBR algorithm is roughly 4.7 times that of a 10 ms RTT flow. In comparison to the BBR, the throughput disparities between other algorithms were smaller, with the BBR-C algorithm having the smallest difference and being relatively stable without significant fluctuations.

Further, evaluate the RTT fairness of the algorithm under different buffer sizes. The Jain fairness index [34] is frequently used to measure the fairness of CCAs, and the calculation is presented in Equation (15). The fairness index, which is based on the data flow’s throughput, can show how equally distributed the bandwidth is between bottlenecks among all active flows.

(15) $$J=\left(\frac{(\sum_{i=1}^{n}x_{i}{)}^{2}}{n\sum_{i=1}^{n}x_{i}{}^{2}}\right)$$

where *x* is the throughput of flow. The Jain fairness index ranges from 0 to 1. The closer the fairness index is to 1, the better the fairness of bandwidth allocation.

**Figure 7:**
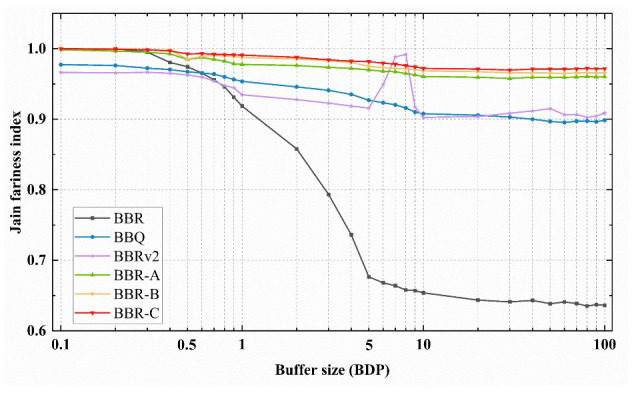
The Jain fairness index of congestion algorithms for 10 ms RTT flows and 50 ms RTT flows competing under different buffer sizes.

We analyze the impact of buffer size on RTT fairness, setting RTT to 10 ms and 50 ms, and varying the buffer size from 0.1 to 100 BDP. Figure 7 displays the Jain fairness index of the algorithm under various buffer sizes. The 50 ms RTT flow consistently outperforms the 10 ms RTT flow, with the throughput gap widening as buffer size increases. Between 5–9 BDP buffer sizes, BBRv2′s 10 msRTT and 50 msRTT flows undergo a throughput crossover, leading to an increase in the fairness index. BBR exhibits the lowest fairness index among the five algorithms while our proposed algorithms maintain a fairness index above 0.964, indicating equal bandwidth sharing between two flows. BBR-C performs best among the three optimization algorithms.

**Figure 8:**
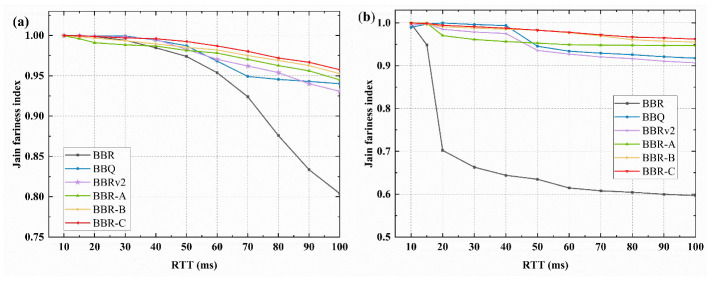
The Jain fairness index when 10 ms RTT flows coexist with different RTTs flows (**a**) 0.5 BDP buffer; (**b**) 5 BDP buffer.

Next, evaluate the impact of the RTT ratio on algorithm RTT fairness under two buffer sizes. Figure 8 depicts the change of the Jain fairness index when a 10 ms RTT flow coexists with different RTTs flows. The RTT fairness of other variant algorithms has improved in different situations as compared to BBR. When the RTT ratio is less than 4, there is no significant difference in the fairness index of the other five BBR variants. Among them, the BBQ and BBRv2 perform well, but their fairness index decreases when the RTT ratio is greater than 4. The changes in BBR-A, BBR-B, and BBR-C algorithms are consistent, with BBR-C maintaining the highest fairness index at roughly 0.965. Overall, the fairness index of BBR-C is the highest among the six algorithms.

**Figure 9:**
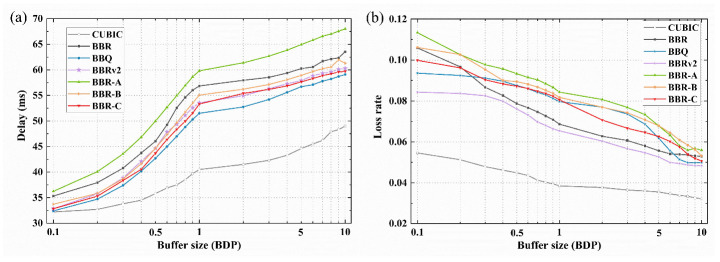
Performance comparison of CCAs in different buffer sizes: (**a**) delay; (**b**) packet loss rate.

Furthermore, the packet loss rate and delay characteristics of several CCAs were compared. A network link with a bandwidth of 100 Mbps and RTT of 30 ms was established. The buffer size ranges from 0.1 to 10 BDP. The delay and packet loss rates are shown in Figure 9. Figure 9a shows that the BBR algorithm and its variants have a longer delay than the CUBIC algorithm, which is around twice. The BBR-A algorithm has the highest latency, up to 66.7 ms. The average delay of the BBRv2 and BBR-C algorithms is close, which is between BBR (56.6 s) and BBQ (52.2 ms). As illustrated in Figure 9b, with the increase in buffer size, the packet loss rate of BBR and its variant algorithms will rapidly decrease to less than 0.1%, with BBRv2 having the lowest packet loss rate. The difference in packet loss rate among these algorithms under different buffer sizes is only 0.015%, and BBR-C is the best among the three optimization algorithms. Overall, the optimization algorithms can effectively adjust the transmission rate under different buffer sizes to achieve better network performance, and BBR-C algorithm synthesis is the best.

### 5.2. Performance Testing in LAN Environments

Configure several network configurations on a local area network test platform, simulate the channel using networking simulation (NetEm) and evaluate RTT fairness. Linux TC (traffic control) to configure different network conditions for real network links, TC-tbf to set the bottleneck buffer size, and the tcpprobe to collect RTT and throughput at the sender. For each network configuration, running the experiment at least 5 times for 200 s each time, the experiment should involve between 2 and 10 flows. Linux TC artificially increases the delay between the sender and receiver (increasing the delay by 10 ms or 50 ms). The bottleneck bandwidth is 100 Mbps, and the buffer size is set to 100 KBytes and 1 MBytes. The selected network parameter ranges such as buffer size, bottleneck bandwidth, and latency are based on commonly used values in modern networks [5,16].

**Figure 10:**
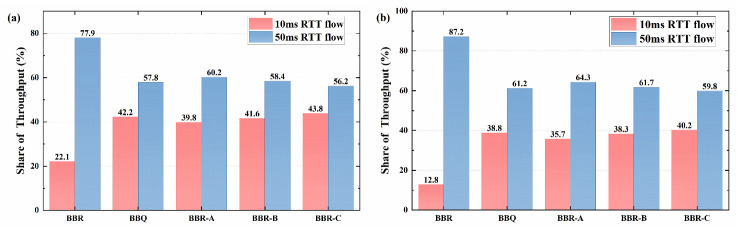
Throughput share of competing data flows in a LAN: (**a**) 100 KBytes; (**b**) 1 MBytes.

Figure 10 presents the statistical results of the throughput ratio of the 10 ms and the 50 ms RTT flow under steady-state conditions. The BBR algorithm and its optimization algorithm, 50 ms RTT flows have significant advantages in different buffer sizes. Although the disadvantages of 10 ms RTT flows have been mitigated in shallow buffers (100 KBytes), their bandwidth share is still significantly lower than it should be. In comparison to the original BBR algorithm, the three improved algorithms based on BBR (BBR-A, BBR-B, and BBR-C) have improved RTT fairness to varying degrees, with the BBR-C algorithm having the highest fairness. Besides, the experimental results of the real network are fully consistent with the simulation outcomes, as observed in the previous simulation tests. Although the actual bandwidth share difference between the two RTT flows in the BBR and BBR-A algorithms is slightly larger than the simulation results, the deviation is only about 1%, which further validates the effectiveness of the simulation experimental results and the optimization algorithm.

### 5.3. Performance of Long-Distance Real Network

Analyze the performance of BBR and its optimization algorithms in long-distance real network scenarios. The cloud server chose the instance path from Beijing to Singapore for network testing and the link with a maximum bandwidth of 10 Gbps.

**Figure 11:**
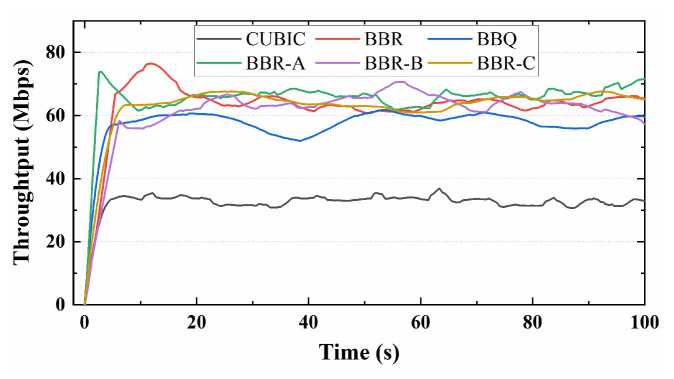
Throughput of congestion control protocols over time in real links.

**Figure 12:**
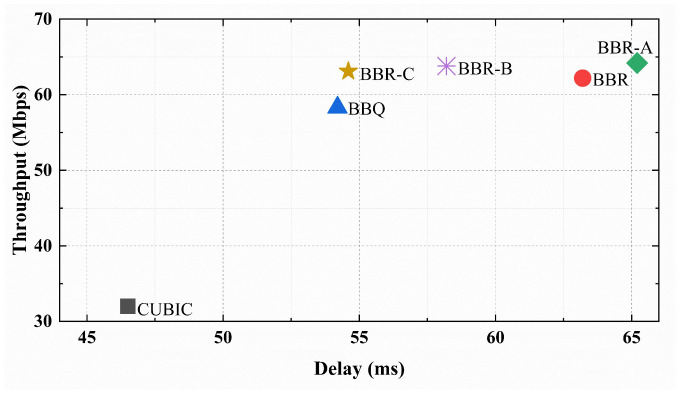
Comparison of throughput and delay in real links.

Firstly, we measured the real throughput variations of several algorithms. The throughput versus time curve over 100 s is depicted in Figure 11. The results indicate that the throughput of BBR and its variant algorithms is significantly higher than that of CUBIC, and the throughput fluctuation of BBR-C is minimal. Subsequently, we conducted tests to assess the average latency and throughput of the algorithms to determine if they can maintain a balance between latency and throughput in long-distance real network links. Figure 12 illustrates that BBR has an average delay of 53 ms, which is twice that of the CUBIC algorithm. Meanwhile, BBR-C exhibits similar latency to BBR but significantly higher average throughput. Overall, in real network scenarios, the BBR-C algorithm has the best performance and can balance throughput and latency.

**Figure 13:**
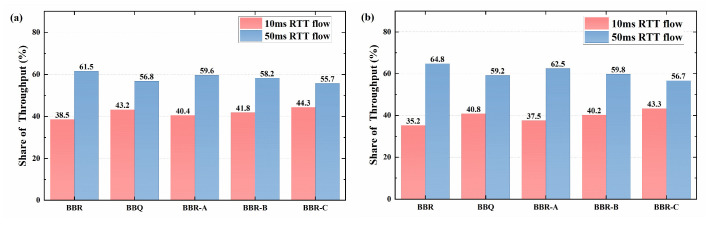
Throughput share of competing data flows in a wide area network: (**a**) 100 KBytes; (**b**) 1 MBytes.

The RTT fairness testing will be carried out in WAN using two links from the lab to the Singapore and Hong Kong node servers, with RTTs of 10 ms and 50 ms, respectively. To facilitate comparison and analysis of the experimental results with local area networks, two buffer sizes will also be configured for the WAN test environment. The statistical results of the throughput share are shown in Figure 13.

The throughput share between 50 ms and 10 ms RTT flows in a wide area network (i.e., RTT unfairness) also exhibits a certain level of deviation, similar to the results of simulation experiments and local area network experiments. This further demonstrates the impact of the RTT ratio on the RTT fairness issue in BBR. However, unlike the obvious benefits of 50 ms RTT flows in LAN, the bandwidth share of 50 ms RTT flows in deep buffers has not significantly increased in WAN. Moreover, the difference in bandwidth usage between the two flows in WAN is also smaller than in simulation experiments and LAN experiments. There are two possible reasons for this result: firstly, there may be a shallow buffer in WAN between the switch and the receiver, which limits the deviation in bandwidth share caused by RTT; secondly, it may be attributed to increased uncertainty in WAN, such as layer by layer routing and processing of data packets by security devices, which makes testing unstable. This may be the main reason why the performance improvement of algorithms measured in real network experiments is slightly lower than the simulation results.

## 6. Conclusions

The primary goal of CCAs is to maintain network fairness. Therefore, starting from the fairness problems inherent in the BBR algorithm, this paper analyzes its detection mechanism and RTT fairness problems, establishes a theoretical model for the existing issues, and proposes pertinent optimization schemes to further improve its congestion control performance.

The impact of fixed pacing gain on RTT fairness is analyzed based on the transmission rate characteristics of the ProbeBW state in the BBR algorithm. To balance the transmission rate between different flows, a pacing gain model was established to adjust the transmission rate nonlinearly. Three gain coefficient models, including the cos function, inverse scale function, and gamma correction curve, are constructed to optimize the BBR algorithm. Based on the BBR algorithm, three optimization algorithms (BBR-A, BBR-B, and BBR-C) are proposed, which successfully enhance the fairness of the BBR algorithm, as demonstrated by performance tests in both simulation and actual network scenarios. Among these algorithms, the BBR-C algorithm provides the best congestion control impact and stabilizes the congestion control process while improving RTT fairness.

This article mainly aims to improve the RTT fairness of BBR. Future work will continue to optimize the model and the selection of key parameters, which could be adaptively adjusted using reinforcement learning and other methods. Additionally, we will keep paying attention to the latest version of BBR and conduct practical application research based on BBR and improved optimization algorithms.

## Data Availability

Not applicable.
